# Pharmacological Activities for *Morus alba* L., Focusing on the Immunostimulatory Property from the Fruit Aqueous Extract

**DOI:** 10.3390/foods10081966

**Published:** 2021-08-23

**Authors:** Bo-Yoon Chang, Bong-Seong Koo, Sung-Yeon Kim

**Affiliations:** 1 Institute of Pharmaceutical Research and Development, College of Pharmacy, Wonkwang University, Jeonbuk, Iksan 54538, Korea; pama611@naver.com; 2 ForBioKorea Co., Ltd., Geumcheon-gu, Seoul 08592, Korea; bskoo@forbio.co.kr

**Keywords:** *M. alba.* L., immunity enhancement, macrophage, review

## Abstract

Depending on the extraction method, numerous compounds that have specific pharmacological effects can be obtained from *M. alba* L. There is a growing scientific interest in health problems related to aging. Efforts to develop safe immune-enhancing pharmaceuticals are increasing. This review aims to summarize and critically discuss the immunity enhancement effects and pharmaceutical efficacy of *M. alba* L. extracts. The scientific database search was conducted using Google Scholar, Web of Science, and PubMed until May 2021. Additional articles were identified and obtained from references in the retrieved articles. Ethanol or methanol extraction of various parts of *M. alba* L. identified a large amount of phenols and flavonoids, which are effective for immunosuppression, antioxidants, and cardiovascular diseases, and are antibacterial, and anticancer. Water extraction of *M. alba* L. enhanced the innate immune response based on immune cell activation. A polysaccharide and an alkaloid related to increased macrophage activity were isolated from *M. alba* L. fruit extracts. *M. alba* L. fruit water extracts primarily induced the production of pro-inflammatory substances, in model organisms, via TLR4 in immune cells. Water extracts have been shown to be effective in pathogen defense and tumor suppression by enhancing macrophage activity. Based on our literature review on the bioactivity of *M. alba* L. fruit extracts, particularly in relation to their immunity enhancement activity, we anticipate that *M. alba*-derived pharmaceuticals will have excellent potential in future medical research.

## 1. Introduction

The individual fruit of the mulberry is small and round, with several fruits grouped together to form a single long oval-shaped panicle. In Korea, the mulberry fruit is sometimes referred to as “Oddi”. A single tree can be very high yielding, containing more than 10 kg of fruit. Mulberry berries are edible and are consumed worldwide, particularly in Asia. Mulberries change color from white to black as they mature and are commonly eaten fresh or processed into foods, such as wine, fruit juices, and jams [1,2,3,4]. Mulberry trees are deciduous plants belonging to the genus *Morus* (family Moraceae), which contains more than 17 species growing in tropical climates. These include *Morus alba* L., *Morus rubra, Morus nigra, Morus australis, Morus atropurpurea, Morus cathayana, Morus notabilis*, and *Morus mesozygia* [5,6,7]. The most common species are *Morus alba* (white mulberry) and *Morus nigra* (blackberry). There has been much research work that has been done since several reviews on the species have been published.

Chen et al. [8] reported that the total phenolic content of mulberry (*M. alba* L. fruits), reaching 502.43 ± 5.10 mg equivalents (QE)/100 g fresh weight (FW), is higher than blackberry, blueberry, raspberry, and strawberry, suggesting that mulberry can be used as good sources of phenolic compounds. Therefore, mulberry fruits are rich in diverse phenolic compounds, including polyphenols, anthocyanins, and flavonoids [9]. As shown in Table 1, *M. alba* (white mulberry) has a higher total dry weight, ash, protein, total carbohydrates, sugar, riboflavin, niacin, total phenols, and alkaloid than *M. nigra* (black mulberry) [10,11] except for pectin [10]. Y. jiang [12] reported that linoleic acid and α-linolenic acid showed higher contents in *M. alba* fruits, about 2.5- and 4-fold, respectively, compared to *M. nigra* fruits. Considering these results, *M. alba* L. contains active ingredients showing high nutritional importance and pharmaceutical effects among the genus *Morus*. *Donguibogam* and *Bencao Gangmu*, *Morus alba* L. have long been used as traditional medicine for diabetes, arthritis, rheumatism, and other disorders for thousands of years in East Asia [13,14,15].

**Figure 1:**
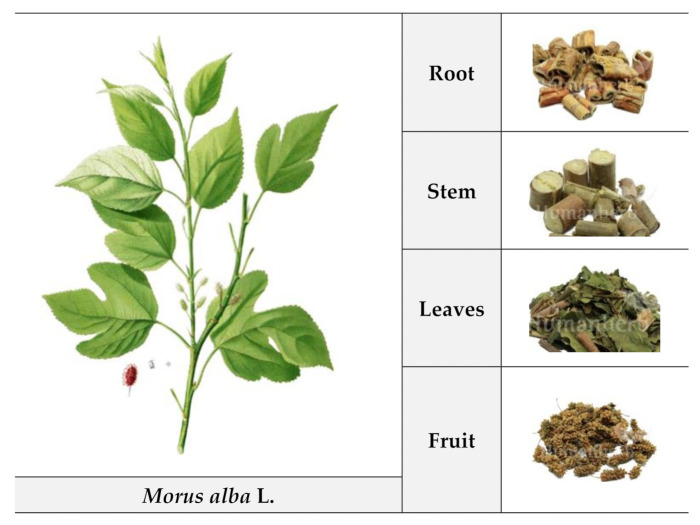
Morphology of *Morus alba* L.

Various parts of *M. alba*, including the roots, stems, leaves, and fruits, have long been used in China, Japan, and Korea as herbal medicines due to their pharmacological effects [18,19,20,21]. Figure 1 illustrates the morphology of *M. alba*. The root bark is called “Sang Bai Pi” and it is used as a diuretic and to treat lung heat and asthma. The dried branch (“Sang Zhi”), collected in spring and summer, is used alone or in combination with other medications to treat rheumatic or rheumatoid arthritis and muscular contractions. The leaves (Sang Ye) have a variety of culinary, medicinal, and industrial applications. They are very palatable and commonly used to make tinctures and herbal teas (a common health beverage in Asian countries). Leaf extracts are thought to cool the blood and stop bleeding and so are used to treat hemoptysis, epistaxis, and hematemesis. The fruits (“Sang Shen”) are thought to enhance liver and kidney function, nourish yin and the blood, and promote the secretion of saliva and moisten dryness [22].

The efficacy of *M. alba* extracts and their active ingredients have been scientifically tested in various cell and animal studies. These studies focused on numerous physiological activities, including the antioxidant, neuroprotective, antiarteriosclerosis, immunomodulation, antitumor, and antihyperlipidemia effects of extracts and active ingredients [2]. Among them, the immunomodulatory effects of *M. alba* extracts are attracting much attention due to an increasing societal interest in delaying ageing and increasing general well-being (e.g., the Lifestyles of Health and Sustainability (LOHAS) demographic) as well as the increasing age profile of certain populations [23,24]. This review summarizes and discusses recent research on the pharmaceutical efficacy of *M. alba* extracts in relation to various functions in the body, in particular immune activity. In this way, we provide information and guidance for future studies on the uses of *M. alba* in treating numerous medical conditions.

## 2. *M. alba* L. Plant Parts: Pharmacological Potential and Bioactive Phytochemical Composition

Root bark, stem bark, leaf, and fruit extracts taken from a single plant species (for medicinal purposes) generally have similar physiological effects on the human body. These may include anti-inflammatory, antioxidant, antidiabetic, antibacterial, anticancer, hepatoprotective, and cardiovascular system protective effects. However, some studies have shown that the physiological effect may differ depending on which plant part the extract was taken from. Table 2 summarizes the known pharmacological effects of *M. alba* plant parts (root, stem, leaves, and fruit).

### 2.1. Root

Methanol extracts of root bark (TRB) of *M. alba* have traditionally been used to treat blood pressure, stabilize blood sugar, and reduce fever [58,103]. Immune-enhancing effects of polysaccharides isolated from the water extract of *Morus alba* L. root [31] but immune-inhibiting effects of Kowanon G [104] and Cudraplavone B [42] isolated from methanol extracts were confirmed. Several studies have identified various phytochemical compounds in the root bark. In 1999, the compound moran 20 K, a glycoprotein with antioxidant and antidiabetic effects, was isolated from methanol extracts [70]. Zheng et al. [104] isolated the compounds moracinoside C, moracin O, and moracin P from water extracts of *M. alba* root bark. Khan et al. [51] investigated the antioxidant activity and phenolic content (total phenols, flavonoids, flavanols and proanthrocyanidins) of methanolic extracts taken from *M. alba* root bark (TRB). TSB showed the highest antioxidant activity then other parts’ extract [51]. These results indicate that there is a high correlation and regression between the phenolic contents and antioxidant potential of these extracts [10]. Ethanol extract from the dried root of *M. australis* (MRE) was also found to decrease CCl_4_-induced hepatic inflammation and necrosis in mice [92].

### 2.2. Stem

Stems extracts have been shown to have anti-inflammatory [37,105,106,107,108] and antiosteoarthritis [109] effects. Ethanol of *M. alba* stem extracts (MSEs) has been been found to suppress IL-6 and IL-8 expression in *Porphyromonas gingivalis* LPS-stimulated hPDL fibroblasts, indicating a possible anti-inflammatory effect. Chen et al. isolated Oxyresveratrol from the methanol of *M. alba* branch extracts [40]. Riviere et al. isolated one new coumarin glycoside, isoscopoletin 6-(6-*O*-β-apiofuranosyl-β-glucopyranoside), with seven known polyphenols from acetone of *M. alba* stem extracts using centrifugal partition chromatography (CPC) [110].

### 2.3. Leaf

The leaves of *M. alba* are used as food for silkworms. They are also one of the most important herbs used in the treatment of hyperglycemia. *M. alba* leaves are also used to treat diabetes mellitus [19,65,66,68,111,112]. According to the investigation by Hunyadi A. [65], an antidiabetic effect of *Morus alba* L. leaves was demonstrated by several iminosugars [113]. Flavonoids and related constituents [114], polysaccharides [70], volatile oil-like fraction of a hot water extract of *M. alba* leaves [62], and ecdysteroids [115] (20-hydroxyecdysone and inokosterone) are thought to play an important pharmcological role [111]. Ecdysteroids are also used in aquaculture of crab and shrimp in addition to their pharmacological effects [116]. Antimicrobial activity [73,74,76] and hepatoprotective effects [49,90] of the ethanol extract of *M. alba* leaves were also observed. The ethanol of *M. alba* leaf and fruit extract (MLFE) supplementation have been found to stimulate cutaneous NLRP3 inflammasomes in HFD-induced obese mice [117]. Chen et al. [28] isolated gamma aminobutyric acid from water extract of *M. alba* leaves by using biochemical methods. The neuroprotective effect of *Morus alba* L. extract is well documented in many studies. Among them, studies on ethanol or methanol extracts from leaves were studied by Chen et al. [96], Yadav et al. [97], and Kang et al. [98]. In terms of the leaf extract, many studies have evaluated the efficacy of the crude extract only, without separating the components.

### 2.4. Fruit

In the history of *M. alba*’s usage in medicine, the fruit has been the last part of the plant to be considered for medicinal purposes. However, fruit phytochemical compounds are now more studied than compounds from other *M. alba* plant parts. Quercetin, pyrrole alkaloids, cyanide, epigallocatechin, epigallocatechin gallate, gallocatechin, gallocatechin gallate, isorhamnetin glucuronide, isorhamnetin hexoside, isorhamnetin hexosylhexoside, kaempferol, glucuronide, kaempferol hexoside, kaempferol hexosylhexoside, kaempferol rhamnosylhexoside, morin, odisolane, and naringin, and phenolic, flavonoid, quinic acid, and anthocyanin compounds have been found in *M. alba* fruits [46,77,78,93,101]. The extract has been used to treat diabetes, arteriosclerosis, hypertension, blood circulation problems, coughing, and asthma in humans, and has recently been scientifically proven to be effective in animal experiments [2,104,117,118]. *M. alba* water extracts (MWEs) have been found to help reduce body weight, serum, and liver lipids in high-fat diet (HFD)-induced obesity [119]. Arfan et al. demonstrated that acetone or methanol extract of *M. alba* have high antioxidant potential, as determined by ABTS, DPPH, and reducing power assays [120]. The fruits of *M. alba*, unlike other parts of the plant, have been more frequently studied in relation to their immunological effects on the human body.

## 3. *M. alba* L. Extraction Solvents and Their Pharmacological Potential

The solvents most commonly used for phytochemical extraction from plant tissue are water, ethanol, methanol, acetone, and ether or a mixture of these [121,122,123]. Table 2 summarizes the pharmacological efficacy of phytochemicals detected in various *M. alba* extract types. It illustrates how efficacy changes depending on solvent type.

Water is used to extract high polar components, such as carbohydrates, amino acids, and glycosides. Ether and acetone are used to separate low polar or aromatic compounds [121,124]. Water extraction is the safest, least expensive, and most environmentally friendly method. Polysaccharides, proteins, polyphenols, and glycosides, which are soluble in water, are separated during water extraction. According to research by Wang et al., ethanol extracts of *M. alba* contained a higher number of phenols and flavonoids than water extracts [26,107]. However, according to Milena et al., *M. alba* water extraction yields more phenolic acids and flavonoids than hydromethanolic extraction methods [125]. Although ethanol or methanol may be selected as a suitable solvent for the separation of active ingredients, such as phenol, plant tissues contain numerous biologically active compounds that require alternate extraction solvents depending on the plant species. In addition, extraction yield is the most important factor in selecting a solvent and is affected by extraction time, temperature, and sample composition, among other factors. Hot water is also used to extract phenolic compounds and polysaccharides at higher concentrations than other solvents, such as ethanol [126]. Research by Peng et al. showed that *M. alba* water extracts (MWEs) contain polyphenols, including gallic acid, chlorogenic acid, rutin, and anthocyanins [119].

Certain endotoxins (LPS) are well-known immunomodulators and often become contaminants in phytochemical extracts. Therefore, several approaches, such as using ultrapure water and filters, are recommended to prevent possible LPS contamination [127,128].

Most plant or fungi extracts that were found to induce immunological activity in the human body were extracted using hot water. In one study on immune activity, the phytochemicals in dried and crushed *M. alba* tissues were extracted using hot water, producing a yield of approximately 25–30% [25,129]. In 2009, Yang’s study was the first to report the immunological activity of phytochemicals extracted using water. This study demonstrated increased phagocytosis, co-stimulatory molecules, cytokine secretion, and MAPK-inase activity in macrophage [130]. Hot water extracts of *M. alba* fruits play an important role in immunity-stimulating activities in the human body.

## 4. Immune Stimulation by Maturity of *M. alba* Fruits

**Figure 2:**
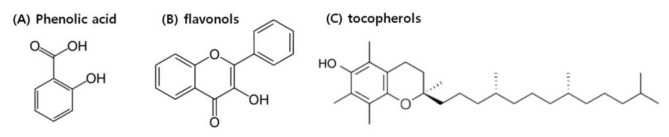
Phytochemicals of immature *Morus alba* L. fruits. Structure of (**A**) phenolic acid, (**B**) flanovols, and (**C**) tocopherols.

The fruits of *M. alba* have been found to induce the highest immune-stimulating effect compared to other plant parts. According to research by Zhang [9], the physical properties of *M. alba* fruits change dramatically during maturation. *M. alba* fruits are classified into four stages: (1) immature, (2) semi-mature, (3) mature, and (4) fully mature. In studies by Liu, mature fruits were found to contain higher total polyphenols and anthocyanins, and also induce higher total antioxidant activity in the model organisms, than immature fruits [131]. Immature fruits have a low pH (which increases storage length). In terms of nutrition, immature fruits have high protein, crude fat, crude fiber, and mineral contents, as well as high concentrations of GABA, amino acids, tocopherols, phenolic acid, and flavonols (which play a functional role in fruit development) (Figure 2).

**Figure 3:**
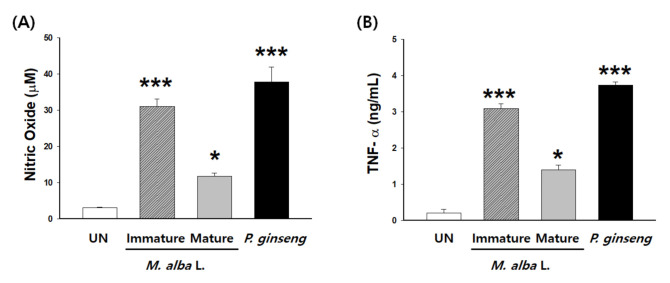
Comparison of murine peritoneal macrophage (RAW 264.7, ATCC^®^ TIB-71™) activity in immature and mature *M. alba* L. fruits and Korean 6-year root red ginseng (KCG, daejeon, Korea). Macrophages were treated with extracts from immature and mature *M. alba* fruits or Korean 6-year root red ginseng extract (100 μg/mL) for 24 h. (**A**) Nitric oxide and (**B**) TNF-α production in supernatants were measured using ELISA. Data are expressed as mean ±SD values. All the data were confirmed by technical replicate 3 times. Significant differences were compared using repeated measures ANOVA followed by the Newman–Keuls multiple range test. Statistical significance was defined as * *p* < 0.05, *** *p* < 0.001. All statistical analyses were performed using GraphPad Software. Inc. (San Diego, CA, USA).

However, no systematic studies have been conducted on how immunity stimulation activity effects on model organisms may change in relation to the extract source in terms of fruit maturity. We conducted an immunological study comparing the effects of water extracts derived from immature versus fully mature fruits. Phytochemicals in fully mature fruits have been extracted using hot water in previous studies [25] and their effects on immune activity in model organisms compared according to fruit maturity (Figure 3). The results for immature fruits were more than three times higher than that of fully mature fruits at the same concentration (100 μg/mL) (without cytotoxicity). Additionally, ginseng, a popular immune stimulator sold in Korea and throughout the world, is produced from hot water crude extracts [132,133] and is sold in the form of beverages, tablets, and capsules [134]. Figure 3 shows our present results, which we measured using the same experimental method as the previous study [25] for comparison with the macrophage activity of Korean 6-year root red ginseng extracts and *M. alba* fruits in the present study. Korean 6-year root red ginseng extracts were purchased from a manufacturer (Junggwanjang KCG, Daejeon, Korea).

The results were equivalent to those reported in the *P. ginseng* experiment and were effective at the same concentrations as those in products that are marketed for immune-enhancing abilities. These results suggest that *M. alba* may be a suitable substitute for ginseng, which currently monopolizes the immunostimulant market.

## 5. Immune Stimulation by *M. alba* Bioactive Phytochemicals

**Figure 4:**
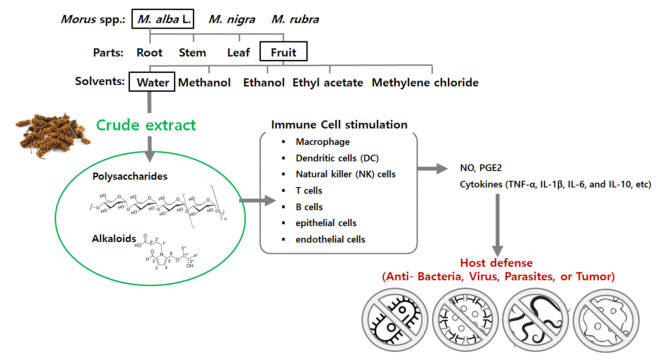
Summary of methods to improve immunological activity of *Morus alba* L.

In *M. alba,* polysaccharides and alkaloids are thought to be the immune-stimulating components and they are more prevalent in the fruits than in any other parts of the plant. Numerous studies have shown that polysaccharides play important roles in various physiological and pathological activities (Figure 4) [135,136].

### 5.1. Polysaccharides

The vast majority of studies that focused on the isolation of *M. alba* polysaccharides and their bioactivities have mainly concentrated on its leaves and fruits, which are medicinal and edible. Among the various parts of *M. alba*, the fruit contains the most polysaccharides. According to a review by Yuan et al., various polysaccharides (FMAP, MFP, MFP-1, MP, etc.) have been isolated and identified in the fruits of *M. alba* through various extraction and purification processes [2]. The composition of polysaccharides varies according to how they are classified. For example, the polysaccharides isolated in a study by Lee et al. [137] were 1639 kDa in mass and were reported as follows: mannose (1.60 mol%), rhamnose (18.40 mol%), glucose (3.10 mol%), galactose (37.60 mol%), xylose (1.70 mol%), fucose (1.30 mol%), and arabinose (36.30 mol%). However, in a study by Chen, the polysaccharides were measured at 13.6 kDa and were reported as follows: rhamnose (25.98 mol%), glucose (13.06 mol%), galactose (23.10 mol%), galacturonic acid (16.35 mol%), and arabinose (21.51 mol%). Therefore, polysaccharides that have different structures may be separated differently and, thus, vary in their efficacy [43,137,138]. For example, Lee et al. [137] observed that a water-soluble polysaccharide (JS-MP-1) induced immunological activity in Raw264.7 cells [139,140]. Similarly, the macrophage immunomodulatory activity of *M. alba*-derived polysaccharides appears to correlate positively with the average molecular weight of these polysaccharides, with the higher molecular weight fractions being the most active [26,139]. Chen et al. reported mulberry leaf polysaccharide (MLP) as a potential mucosal vaccine adjuvant candidate against ND in chickens [30].

Immune stimulation in the defense against diseases in humans is currently receiving much attention. In particular, there is a growing interest in mushroom polysaccharides as well as various fruit polysaccharides (FPs), and studies are underway to identify these polysaccharides and their biological activities in model organisms. Shin et al. [7] reported that an *M. alba* polysaccharide can be used as an adjuvant in dendritic cell-based cancer immunotherapy, as it induces phenotypic maturation of dendritic cells [7].

### 5.2. Alkaloids

**Figure 5:**
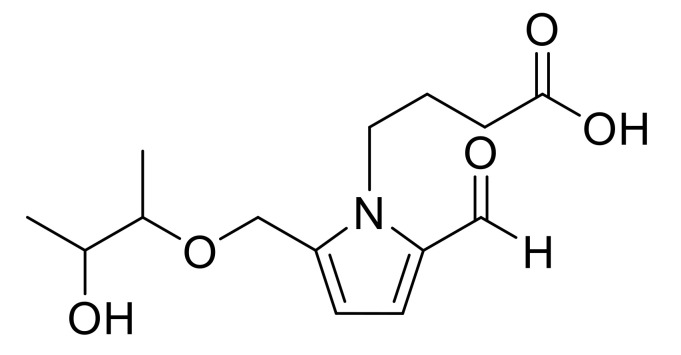
Structure of morrole A from *Morus alba* L. fruits.

Kim et al. [27] separated several alkaloids from *M. alba* using spectroscopic data interpretation. Among them, morrole A (Figure 5), first isolated from *M. alba* fruit, has been reported to cause an increase in nitric oxide, TNF-α, and IL-12 production, as well as phagocytosis, through increased macrophage activity in RAW264.7 cells. However, with plant extracts, it is difficult to separate a single component in order to understand its efficacy in model organisms because various components have shown complex efficacies.

## 6. The Mechanism of Action of *M. alba* Fruit Extract on the Immune System

### 6.1. The Immune Stimulation

*M. alba* fruit water extracts primarily induce the production of inflammatory substances, in model organisms, via TLR4 in immune cells [25,129]. Toll-like receptors (TLRs) are a group of PRRs found in immune cells (macrophage cells, dendritic cells (DCs), natural killer (NK) cells, T cells, B cells, epithelial cells, and endothelial cells). Muscle cells and adipocytes also play important roles in pathogen recognition and inducing an immune response. TLR stimulation mainly activates the innate immune response [141,142]. Unregulated TLR activity increases the risk of developing chronic inflammatory and autoimmune diseases. Currently, various natural compounds and their derivatives have been found to act as agonists or antagonists to TLR family members and their downstream signal transduction molecules [143]. Research on the relationship between plant phytochemical extracts and TLR4 is increasing. The activation of TLR4 by *M. alba* activates MAPK-inase, which in turn increases NF-κB-induced expression of pro-inflammatory factors, such as TNF-α, IL-1β, and IL-6. According to a recent study by Chang et al. [25], the effects of *M. alba* extracts on macrophage NO and TNF-α production were also inhibited by anti-TLR4 antibodies. Moreover, *M. alba* extracts failed to induce production of NO and TNF-α in peritoneal macrophages obtained from C3H/HeJ mice, which have a point mutation in the TLR4 gene. This suggests that the TLR4 molecule is involved in NO and TNF-α production-mediated macrophage activation.

### 6.2. Host Defense

Water extracts of *M. alba* fruits have not been found to be cytotoxic to colon, bladder, breast, or liver cancer cells (in vitro). Chang et al. explored the synergistic antitumor effects of MFE and a drug called 5-fluorouracil in a CT26 cell xenograft model. Leukocyte counts, spleen weight, NK cells, and CTL activity in the tumor xenograft mice significantly increased in the MFE/drug group [25]. In addition, MFE was not found to have any antibacterial effects on *S. typhimurium* or *E. coli*. However, an in vivo study showed that oral administration of MFE for five days significantly increased survival in salmonella-infected mice [129]. The antitumor and antibacterial activity of MFE is thought to be a result of immune-stimulatory effects. Approximately 15% of all circulating lymphocytes are NK cells that can lyse cancer cells in vitro without prior immunity sensitization. Their main function is early host defense against both allogenic and autologous cells following infection by a virus, bacteria, or parasites or the growth of tumor cells [144,145].

### 6.3. Inflammatory Response

The macrophage is one of the most important innate immune cells in the body. It is present in various forms in all tissues. In addition, the action of these macrophages is known to be an important component of major immune responses, such as adaptive immunity, wound healing, and the inflammatory response [146,147,148]. The morphologies and functions of macrophages vary widely. They circulate through the blood and reside in tissues. They are known as microglial cells in the brain or Kupffer cells in the liver [149]. The activities of both types of macrophages are regulated by interaction with adjacent cells. In turn, activated macrophages affect the adjacent environment [150]. Therefore, macrophages perform important functions as part of homeostatic regulation of the body. It is suspected that abnormalities in the function of these macrophages may cause various diseases. Therefore, studies are currently being conducted in order to better understand macrophage activity. It is also important to identify drugs that can regulate macrophages. Various crude extracts of *M. alba* have been observed to exhibit favorable pharmacological effects due to their ability to modulate macrophage function. Kwon et al. found that a water extract of *Mori folium* (WEMF) significantly stimulated the production of NO and PGE2 as immune response parameters at non-cytotoxic concentrations. This was associated with increased expression of inducible NO synthase and COX-2. The release and expression of cytokines, such as TNF-α, interleukin (IL)-1β, IL-6, and IL-10, also significantly increased in response to treatment with WEMF [151]. Yang [130] and Chang et al. [25] observed that crude extracts of *M. alba* fruits induced potent macrophage immunomodulatory activity, as demonstrated by an induction of effector molecules, such as NO and cytokines. In addition, treatment with crude extracts of *M. alba* before PMA resulted in a significantly enhanced response, which is indicative of a priming effect. Many immunomodulatory compounds, including LPS, can prime phagocytes for enhanced ROS production, and it is generally thought that priming plays a key role in the host-defense process and may be essential for host survival against microbial pathogens [125,139,152].

## 7. Safety of *M. alba* Extracts

Toxicity of herbal products is often reported in consumers as a result of incorrect methods of administration, particularly overdoses [153]. Therefore, traditionally used herbal products must be consumed in non-toxic doses that are scientifically verified.

*M. alba* has long been consumed fresh (i.e., fruits) and in traditional medicine products. Its biological activities have been well studied. The intra-gastric administration of ethanolic extracts of *M. alba* fruits to rats at a maximum dose of 1000 mg/kg was not found to cause changes in their behavior, such as respiratory changes, weight loss, or death, within one week of continuous administration. This indicates that this extract type at the stated dose does not have any acute toxicity effects [118]. Juhas et al. orally administered Sprague-Dawley (SD) rats with MFE (*M. alba* fruit water extract) at the highest concentration of 2000 mg/kg, but no toxicity effects were observed [146,154]. In a study by Marx, no toxicity effects on male and female SD rats were observed following administration of an oral dose for 28 days at concentrations of 4000 mg/kg [155]. Peng et al. studied the toxicity of MWE (*M. alba* fruit water extract) on hamsters by examining the effects of oral administration (0.5–2%, *w*/*w*) of the extracts for 12 weeks [119]. Chang et al. investigated the sub-chronic oral toxicity and genotoxicity of MFE. SD rats were treated with MFE (*M. alba* fruit water extract) daily using oral doses at 40, 200, and 1000 mg/kg concentrations for 90 days [156]. The effect level at which no adverse effect was observed in this subacute toxicity study was considered to be 1000 mg/kg, the highest dose tested. In another study, no *Salmonella typhimurium* strains (TA98, TA102, and TA1535) treated with MFE exhibited genotoxicity. The effects of ingesting *M. alba* fruits were examined in health risk tests on adult humans and the results showed that there were no significant carcinogenic risks associated with consumption of *M. alba* fruits. Therefore, the consumption of *M. alba* fruits is considered to be safe.

## 8. Conclusions

*M. alba* L. products are rich in nutrients and bioactive compounds, and have a variety of pharmacological properties, which may help prevent or treat chronic diseases. *M. alba* L. contains more phenolic compounds than other berries, and more riboflavin, niacin, total phenols, and alkaloid than other mulberry species. In order to enhance the immunological activity of *M. alba* L., it is effective to extract immature fruits with water. Polysaccharides and pyrrol alkaloids are known for their macrophage-activating effects in *M. alba* L. In the crude extract extracted with water, it is considered that unknown components act in combination on immune enhancement. Therefore, there is a need for further research on unidentified compounds in *M. alba* fruits and their biological effects.It is also important to develop consistent phytochemical profiles for consumption and clinical trials. To date, there is little information on the metabolomics of phytochemicals, such as alkaloids and polysaccharides. Therefore, it is important to investigate the metabolites formed in the body and how they exert their immunopotentiating effects.

## Figures and Tables

**Table 1:** Proximal composition, sugar, pectin, total phenol, and alkaloid of two species of mulberry.

Composition	*M. alba* (White Mulberry)	*M. nigra* (Black Mulberry)	Reference
Ash (mg/100 g DW)	0.57 ± 0.11	0.50 ± 0.08	[10,16]
Protein(mg/100 g DW)	1.55 ± 0.30	0.96 ± 0.16	[10]
Total sugar (g/100 g FW)	7.55 ± 1.01	6.64 ± 1.12	[10]
Reducing sugar (g/100 g FW)	5.90 ± 0.92	4.94 ± 0.73	[10]
Pectin (g/100 g FW)	Not detected	0.76 ± 0.03	[10]
Riboflavin (g/100 g FW)	0.88 ± 0.00	0.04 ± 0.00	[10]
Niacin (mg/100 g FW)	3.10 ± 0.60	1.60 ± 0.10	[10]
Total phenols (mg/100 g FW)	1650 ± 12.25	880 ± 7.20	[10,11]
Alkaloid (mg/100 g FW)	660 ± 5.25	630 ± 5.93	[10]
Total anthocyanins content(C3G µg/g frozen weight)	911.8	719	[17]

(DW: dry weight; FW: fresh weight).

**Table 2:** Studies that investigated the pharmacological effects of phytochemicals present in *M. alba* L. extracts (grouped according to different parts or extract solvent types).

Pharmacological Effect	Part	Solvents	Extract or Phytochemical	Reference	Type (Species)
**Immune enhancing**	Fruit	Water	Crude extract	[25]	in vitro and in vivo (mouse)
			Polysaccharide	[7,26]	in vitro
		Methanol	Pyrrol alkaloid Morrole A	[27]	in vitro
	Leaf	Water	Polysaccharide	[28,29,30]	in vitro and in vivo (mouse, chicken)
	Root	Water	Polysaccharide	[31]	in vitro
**Immune inhibiting**	Fruit	Ethanol	Crude extract	[32]	in vitro
	Leaf	Ethanol	Crude extract	[32,33,34,35]	in vivo
		Methanol	Crude extract	[36]	in vivo (mouse)
	Stem	Ethanol	Crude extract	[37,38,39]	in vitro
			Oxyresveratrol	[40]	in vitro
	Root	Methanol	Kuwanon G	[41]	in vivo (mouse)
			Cudraflavone B	[42]	in vivo (mouse)
**Antioxidant**	Fruit	Water	Polysaccharide	[43,44]	in vitro
		Ethanol	Crude extract	[45]	in vitro
		Methanol	Hydroxycinnamic acid esters, Flavonol glycosides, andAnthocyanins	[46]	in vitro
		Ethyl acetate	Crude extract	[47]	in vitro
	Leaf	Water	Polysaccharide	[48]	in vitro
		Ethanol	Crude extract	[29,34,49,50]	in vitro
	Root	Methanol	Phenolic contents	[51]	in vitro
**Cardiovascular system protection**	Leaf	Ethanol	Crude extract	[52,53]	in vitro, in vivo (Rat)
		Ethyl acetate	Crude extract	[54]	Ex vivo (Rat)
		petroleum ether, dichloromethane, ethanol	Crude extract	[55]	in vitro
	Root	Water	Moracinoside C, Moracin O, Moracin P	[56]	in vitro
		Ethanol	Crude extract	[57]	in vitro, in vivo (Rat)
		Methanol	Crude extract	[58]	in vitro
			Morusinol	[59]	in vitro, in vivo (Rat)
**Antidiabet** **ic**	Fruit	Water	Crude extract	[43]	in vitro
		Ethanol	Crude extract	[47,60]	in vitroin vivo (Rat)
		Methanol	Crude extractantocyanins	[61]	in vitro, in vivo (Mouse)
	Leaf	Water	Crude extract	[62,63,64]	in vitro, in vivo (Mouse)
		Ethanol	Crude extract	[34,65,66,67]	in vitro, in vivo (Rat, Mouse)
		Methanol	chlorogenic acid, rutin, isoquercitrin loliolide,1-deoxynojirimycin, fagomine 2-O-alpha-D-galactopyranosyl-1-deoxynojirimycin	[68]	in vitro, in vivo (Rat, Mouse)
	Root	Ethanol	Crude extract	[69]	in vitro, in vivo (Rat)
		Methanol	Moran K	[70]	in vitro
		Ethyl acetate	Crude extract	[71]	in vitro
**Antibacterial**	Fruit	Water	Crude extract	[72]	in vitro
	Leaf	Ethanol	Crude extract	[73,74,75]	in vitro, in vivo (Rat)
			1-deoxynojirimycin	[76]	in vitro
	Stem	Ethanol	Crude extract	[38]	in vitro
**Anticancer**	Fruit	Ethanol	Butyl pyroglutamate quercetin, 3-*O*-β-D-glucoside kaempferol, 3-*O*-β-D-rutinoside, rutin, and 2-phenylethyl d-rutinoside	[77]	in vitro
			Odisolane	[78]	in vitro
	Leaf	Ethanol	Flavonoid	[79,80]	in vitro
		Methanol	Morin	[81]	in vitro
	Root	Methylene chloride	Crude extract	[82]	in vitro
		Methanol	Crude extract	[83]	in vitro
			Albanol A	[84]	in vitro
			Morusin	[85]	in vitro
**Hepato** **-protection**	Fruits	Water	Polysaccharide	[86]	in vitro
		Chloroform	Benzofuran, Isomoracin, N-(N-benzoyl-l-phenylalanyl)-l-phenylalanol	[87]	in vitro
	Leaf	Water	Crude extract	[88,89]	in vivo (Rat)
		Ethanol	Crude extract	[49,90]	in vivo (mouse)
		Methanol	Crude extract	[91]	in vivo (mouse)
	Root	Ethanol	Crude extract	[92]	in vitro and in vivo (mouse)
**Neuroprotection**	Fruit	Ethanol	Flavonoid	[93,94,95]	in vitro and in vivo (mouse)
	Leaf	Ethanol	sesquiterpenoid glucoside, aromatic glucoside, farnesylacetone derivative, flavan, and (9R)-hydroxyl-(10E, 12Z,15Z)-octadecatrienoic acid	[96]	in vitro
		Methanol	Crude extract	[97,98]	in vivo (mouse)
		Acetone	Crude extract	[99]	in vivo (Rat)
	Root	Methanol	mulberrofuran G, albanol B, kuwanon G	[100]	in vitro
**Antiobesity**	Fruit	Water	Pyrrol alkaloid	[101]	in vitro
	Leaf	Water	Crude extract	[102]	in vivo (mouse)
		Ethanol	Crude extract	[49]	in vivo (mouse)

## Data Availability

The data generated during this study are included in this article and are available on request from the corresponding author.
